# NFATc2-dependent epigenetic upregulation of CXCL14 is involved in the development of neuropathic pain induced by paclitaxel

**DOI:** 10.1186/s12974-020-01992-1

**Published:** 2020-10-18

**Authors:** Meng Liu, Su-Bo Zhang, Yu-Xuan Luo, Yan-Ling Yang, Xiang-Zhong Zhang, Bo Li, Yan Meng, Yuan-Jie Chen, Rui-Xian Guo, Yuan-Chang Xiong, Wen-Jun Xin, Dai Li

**Affiliations:** 1 Neuroscience Program, The Fifth Affiliated Hospital of Sun Yat-Sen University, Guangdong Province Key Laboratory of Brain Function and Disease, Sun Yat-Sen University, Guangzhou, 510080 China; 2 Department of Hematology, The Third Affiliated Hospital, Sun Yat-Sen University, Guangzhou, 510630 China; 3 Department of Anesthesiology, Changhai Hospital, Naval Medical University, 168 Changhai Road, Shanghai, 200433 China; 4 Department of Physiology and Pain Research Center, Zhongshan Medical School, Sun Yat-Sen University, 74 Zhongshan Road 2, Guangzhou, 510080 China

**Keywords:** Paclitaxel, NFATc2, CXCL14, Histone acetylation, Neuropathic pain

## Abstract

**Background:**

The major dose-limiting toxicity of paclitaxel, one of the most commonly used drugs to treat solid tumor, is painful neuropathy. However, the molecular mechanisms underlying paclitaxel-induced painful neuropathy are largely unclarified.

**Methods:**

Paw withdrawal threshold was measured in the rats following intraperitoneal injection of paclitaxel. The qPCR, western blotting, protein or chromatin immunoprecipitation, ChIP-seq identification of NFATc2 binding sites, and microarray analysis were performed to explore the molecular mechanism.

**Results:**

We found that paclitaxel treatment increased the nuclear expression of NFATc2 in the spinal dorsal horn, and knockdown of NFATc2 with NFATc2 siRNA significantly attenuated the mechanical allodynia induced by paclitaxel. Further binding site analysis utilizing ChIP-seq assay combining with gene expression profile revealed a shift of NFATc2 binding site closer to TTS of target genes in dorsal horn after paclitaxel treatment.

We further found that NFATc2 occupancy may directly upregulate the chemokine CXCL14 expression in dorsal horn, which was mediated by enhanced interaction between NFATc2 and p300 and consequently increased acetylation of histone H4 in CXCL14 promoter region. Also, knockdown of CXCL14 in dorsal horn significantly attenuated mechanical allodynia induced by paclitaxel.

**Conclusion:**

These results suggested that enhanced interaction between p300 and NFATc2 mediated the epigenetic upregulation of CXCL14 in the spinal dorsal horn, which contributed to the chemotherapeutic paclitaxel-induced chronic pain.

**Supplementary information:**

The online version contains supplementary material available at 10.1186/s12974-020-01992-1.

## Background

Paclitaxel, as a first-line antitumor drug, was widely used to treat solid tumor [1]. Paclitaxel generally binds to β-tubulin and impairs axoplasmic transport, thereby leading to a progressive, dying-back neuropathy. The painful neuropathy was the major reason for the delay or discontinuation of chemotherapy [2]. However, the central mechanism underlying paclitaxel-induced chronic pain is still largely unclear.

NFAT (nuclear factor of activated T cell) protein is first discovered in T cells as transcriptional activators of IL-2 [3]. Calcineurin-mediated NFAT dephosphorylation triggers the NFAT protein translocated into the nuclear, which subsequently functions as a transcriptional factor to regulate the expression of target genes [4]. There are four classic members in the NFAT gene family: NFATc1, NFATc2, NFATc3, and NFATc4 [5]. Among them, NFATc2 is an important inflammation-associated transcription factor and participated in various pathological processes in the central nervous system [6, 7]. Activation of NFATc2 regulates the expression of several cytokines including IL-2 or IL-12, by which it is critically involved in the regulation of immunity of the host [8, 9]. Accumulating evidence also showed that NFATc2 is widely expressed in the CNS and plays critical roles in neurological diseases [10, 11]. For example, the activation of calcineurin A/NFATc2 pathway in the hippocampus was associated with the degree of dementia in AD patients [12]. Furthermore, study showed that the NFATc4 expression in DRG contributed to the chronic pain after nerve injury [13]. However, whether the NFATc2 in spinal dorsal horn is involved in chronic pain induced by chemotherapeutic drug paclitaxel remains unknown.

It is well-known that imbalance between pro-nociceptive cytokines and anti-nociceptive cytokines play an important role in the initiation and maintenance of chronic pain. The tissue damage induces the activation of the CNS immune cells such as microglia and astrocyte, which synthesized and released the pro-nociceptive cytokine and contribute to the chronic pain [14, 15]. Considerable data exist to indicate that chemokines can act as pro-nociceptive mediators following tissue injury and disease in the nervous system [7]. For instance, the upregulation of chemokines including CXCL12, CX3CL1, and CCL2 in the nervous system is involved in the chronic pain induced by nerve injury or chemotherapeutic drugs [16–18]. Among chemokines, CXCL14, as an evolutionary ancient chemokine, is constitutively expressed in the brain and other tissues [19]. To date, CXCL14 receptor has not yet been identified, and its function remains known. While anecdotal evidence shows the upregulation of CXCL14 following the nerve injury [20], whether CXCL14 contributes to the chronic pain induced by paclitaxel treatment remains unknown. Furthermore, whether transcript factor NFATc2, as an inflammation-associated transcription factors, regulated the expression of CXCL14 is not reported.

## Methods

### Animals

Sprague-Dawley male rats (220–260 g) were obtained from the Institute of Experimental Animals in Sun Yat-Sen University. The animals were kept at room temperature with 50 to 60% humidity and were housed in the separated cages with ad libitum access to food and water. All animals were randomly assigned to different experimental or control conditions in the present study. All experiment procedures were approved by the Local Animal Care Committee and were performed in accordance with the guidelines of the National Institutes of Health on animal care and the ethical guidelines.

### Drug administration and behavioral test

We previously found that treatment with moderate-dose paclitaxel (cumulative dose of 24 mg/kg in 3 × 8 mg/kg), which was adopted from doses applied in clinical patients establish a stable model of neuropathic pain with remarkable mechanical allodynia [21]. Paclitaxel (Taxol, Bristol-Myers Squibb, 6 mg/ml, New York, NY) was diluted with saline (1:3) in this study. Solution of paclitaxel was prepared before each application and injected intraperitoneally (8 mg/kg/day) on days 1, 4, and 7 at 9:00 AM. Some volume of saline was injected at the same schedule in the control group. NFATc2 siRNA (50 μg/15 μl, Ribobio), CXCL14 siRNA (50 μg/15 μl, Ribobio), FK506 (10 μg/10 μl), or scrambled siRNA was initiated 30 min before the first dose of paclitaxel and maintained for 10 consecutive days.

For behavior test, all animals in this project have been pre-tested. Each group of animals was placed in a plastic box for 3 consecutive days, 15 min per day in order to allow them to adapt to the environment before testing. Mechanical allodynia tests were assessed using Von Frey hairs. Von Frey filaments that produce different forces were applied alternately to the plantar surface of hind paw. The up-down method was used according to the peer’s report [22]. In the absence of a paw withdrawal response, a stronger stimulus was presented. When paw withdrawal occurred, the next weaker stimulus was chosen. Optimal threshold calculation by this method required 5 responses in the immediate vicinity of the 50% threshold [16, 22]. Thermal hyperalgesia was tested using a plantar test (Ugo Basile Plantar Test Apparatus). Briefly, a radiant heat source beneath a glass plate was aimed at the plantar surface of the hind paw. Three measurements of hind paw withdrawal latency were taken for each hind paw and averaged as the result of each test. A 25-s cutoff was set to prevent tissue damage. All experiments were performed by investigators who were blinded to the treatments/conditions.

Intrathecal injection was preformed according to the method previously described [23, 24]. In brief, a polyethylene-10 catheter was implanted into the L5/L6 intervertebral subarachnoid space; the localization of the tip of the catheter was between the levels of the L4–L6 spinal segments. Animals were allowed to recover for 5 days. Animals, which exhibited hind limb paresis or paralysis after surgical operation, were excluded from the experiment. The intrathecal injection was performed using aseptic techniques. siRNAs or FK506 were intrathecally injected 30 min prior to paclitaxel or saline treatment in the animals under isoflurane (4%) anesthesia.

### Immunohistochemistry

Perfusion was performed through the ascending aorta with 4% paraformaldehyde after an application of sodium pentobarbital at 50 mg/kg dose (i.p.). The lumbar segments of the spinal cord were removed and placed into 4% paraformaldehyde for 3 h, and stored in 30% sucrose overnight. Cryostat sections (16 μm) were cut and processed for immunohistochemistry with primary antibodies for CXCL14 (1:200; Abcam, rabbit), NFATc2 (1:200, Thermo Fisher Scientific, mouse), Iba1 (1:25, Sigma, goat), NeuN (1:200, Chemicon, rabbit), GFAP (1:200, Chemicon, rabbit), and Sox10 (1:1000, Thermo, rabbit). Absence of primary antibody was applied as the negative control. After incubation overnight at 4 °C, the sections were incubated with secondary antibodies, which conjugated with cy3 or fluorescein isothiocyanate for 2 h at RT. The stained sections were then examined with a Leica (Leica, Germany) fluorescence microscope, and images were captured with a Leica DFC350 FX camera. NIH ImageJ was used to quantify the intensity of immunofluorescence statistical analysis. An intensity threshold was set above background level firstly to identify structures with positive staining signals. The area occupied by these structures was measured as positive area. In each rat, four to six sections (25 μm thickness) of the spinal cord were selected randomly. The ratio of NFATc2 intensity to DAPI intensity represented co-staining and was obtained in each animal across the different tissue sections. Then, the mean ± SE across animals was determined in each group.

### Western blot

Animals were anesthetized with sodium pentobarbital (50 mg/kg, i.p.) at various time points. L2–S1 segments were separated and L4–L6 segments were chosen for the following analysis. Five hundred-micrometer-thick acute spinal L4–L6 cord slices were cut on a vibratome (Leica VT-1000 S) in standard artificial cerebrospinal fluid with continuous oxygenation. Dorsal horn tissue was punched with a 15-gauge cannula from the slices in a cryostat, and then homogenized in 15 mmol/l Tris buffer containing a cocktail of proteinase inhibitors and phosphatase inhibitors on ice. Nuclear and cytoplasmic extracts were prepared at the desired time points using NE-PER nuclear and cytoplasmic extraction reagent (Thermo Scientific). Protein samples were separated by gel electrophoresis (SDS-PAGE) and transferred onto a PVDF membrane. The membrane was placed in the blocking buffer for 1 h at room temperature and incubated with primary antibodies against CXCL14 (1:1000; Abcam), NFATc2 (1:1000, Santa Cruz), acetylated histone H3 (1:1000, Millipore), acetylated histone H4 (1:1000, Millipore), histone H3(1:1000, Millipore), or β-actin (1:2000, Cell Signaling Technology) overnight at 4 °C. Then, the blots were then incubated with horseradish peroxidase-conjugated secondary antibody for 2 h at RT. ECL (Pierce, USA) was used to detect the immune complex. The bands were quantified with a computer-assisted imaging analysis system (NIH ImageJ).

### siRNA preparation and screening

Specific siRNAs were used to knockdown the expression of CXCL14 and NFATc2. Three siRNA-targeting rat CXCL14 gene or NFATc2 gene were designed and synthesized by Ribobio (Guangzhou, China) for the subsequent experiments, respectively. The siRNA sequence of CXCL14 gene and NFATc2 gene is shown in Supplemental Table 1. According to the previous screening test, siRNAs were transfected into the HBZY-1 cells using Lipofectamine 2000 (Invitrogen, Carlsbad, CA). The siRNA, which has no homology to CXCL14 or NFZTc2 gene, was used as control (Scramble). The expression of CXCL14 mRNA was suppressed by 87.77 ± 6.19%, 33.92 ± 7.56%, and 59.90 ± 6.08% in cell lines when treated with CXCL14 siRNA1, 2, and 3, respectively. The expression of NFATc2 mRNA was suppressed by 87.85 ± 7.53%, 57.84 ± 8.33%, and 28.05. ± 9.65% in cell lines when treated with NFATc2 siRNA1, 2, and 3, respectively. Consistent with the suppression of mRNA, the CXCL14 and NFATc2 protein levels were remarkably reduced after CXCL14 siRNA1 or NFATc2 siRNA1 treatment (Supplemental Figure. 1). Therefore, the chemically synthesized CXCL14 siRNA1 and NFATc2 siRNA1 were chosen for the subsequent experiments in vivo.

### RNA extraction and real-time qPCR

Trizol was used to extract total RNA from dorsal horn tissues. The reverse transcription was performed according to the manufacturer’s protocol of polymerase chain reaction (PCR) production kit. The primer sequences for PCR assay on all targeted mRNAs are presented in Supplemental Table 2. Real-time qPCR was performed using SYBR Green qPCR SuperMix (Invitrogen) and the ABI PRISM7500 Sequence Detection System. The reaction conditions included incubation at 95 °C for 3 min followed by 40 cycles of thermal cycling (10 s at 95 °C, 20 s at 58 °C, and 10 s at 72 °C). The ratio of mRNA expression was analyzed by the 2^−ΔΔCT^ method.

### Coimmunoprecipitation

Coimmunoprecipitation (Co-IP) was carried out using the Coimmunoprecipitation Kit (Pierce, Rockford, IL). Briefly, spinal dorsal horn tissues were excised quickly and put into lysis buffer. The p300 antibody or NFATc2 antibody, which was immobilized with resin, was used to collect the immune complexes. The eluted complexes from the resin were analyzed by western blot using NFATc2 antibody or p300 antibody after incubation and washes.

### Chromatin immunoprecipitation

Chromatin immunoprecipitation (ChIP) assays were performed using the ChIP Assay Kit (Thermo) as described previously [17]. The animal’s L4 and L5 spinal cord was removed quickly and placed in 1% formaldehyde for 10 min at room temperature to cross-link transcription factors with chromatin. The formaldehyde was then inactivated by addition of 125 mM glycine. Sonicated chromatin extracts containing DNA fragments were immunoprecipitated using 6 μg of ChIP-grade NFATc2 antibody or normal rabbit IgG antibody with pre-blocked protein G-Sepharose beads overnight at 4 °C. The next day, the chromatin-protein-antibody-bead complexes were eluted, and the DNA was extracted. The precipitated DNA was resuspended in the nuclear-free water, and qPCR assays with primers (Supplemental Table 3) were performed to amplify the different region within the CXCL4 promoter, containing the NFATc2 motif (red font). Finally, the ratio of ChIP/input in the spinal dorsal horn was calculated.

### ChIP-seq identification of NFATc2 binding sites

ChIP-seq libraries were prepared from a total of 10 ng DNA using TruSeq Nano DNA Sample Prep Kit (Illumina) according to the manufacturer’s instructions. The completed libraries were quantified by 2100 Bioanalyzer (Agilent, Waldbronn, Germany). The libraries were then sequenced by running 2 × 150 cycles on the Illumina HiSeq 4000 following the HiSeq 3000/4000 SBS Kit protocol (Illumina). After the sequencing platform generated the sequencing images, the stages of image analysis and base calling were performed using the Off-Line Basecaller software V1.8. Sequence quality was examined using the FastQC software. After passing Solexa CHASTITY quality filter, the clean reads were aligned to Rat genome (UCSC RN5) using the BOWTIE software V2.1.0 [25]. The MACS V1.4.2 program [26] was then used for peak calling of the ChIP enrichment regions relative to control data set that was generated from input samples. The peaks in samples were annotated by the nearest gene using the newest UCSC RefSeq database.

### Microarray analysis

Total RNAs were reverse transcribed into double-stranded cDNAs. Then cDNAs in vitro transcribed into antisense cRNAs and labeled with Cy3-CTP and Cy5-CTP using a Two-Color Low Input Quick Amp Labeling Kit (Agilent Technologies, Santa Clara, CA, USA). Fluorescence dye-labeled cRNAs were fragmented and hybridized on Sure Print G3 Rat GE 8x60K Microarray using an Agilent Gene Expression Hybridization Kit. The fluorescence intensities at 635 nm (Cy5) and 532 nm (Cy3) were scanned by an Agilent microarray scanner. Microarray data were extracted using the Agilent Feature Extraction Software. Low intensity spots were removed, and each gene had expression value in more than 80% samples analyzed. Signals were normalized by Loess normalization. Differentially expressed genes (DEGs) were screened using the SAM v4.01 software, with the false discovery rate set to 5% (*q* value < 0.05) [27].

### Statistical analysis

All data were expressed as the means ± SEM and analyzed with SPSS 22.0 (SPSS, USA). Western blot and qPCR data were analyzed with two-way analysis of variance (ANOVA) followed by a Tukey post hoc test. For behavioral tests, one-way or two-way ANOVA with repeated measures followed by a Tukey post hoc test was carried out. The criterion for statistical significance was *P* < 0.05. While no power analysis was performed, the sample size was based on previous studies of painful behavior and pertinent molecular studies.

## Results

### Upregulation of NFATc2 contributed to the mechanical allodynia induced by paclitaxel treatment

**Fig. 1:**
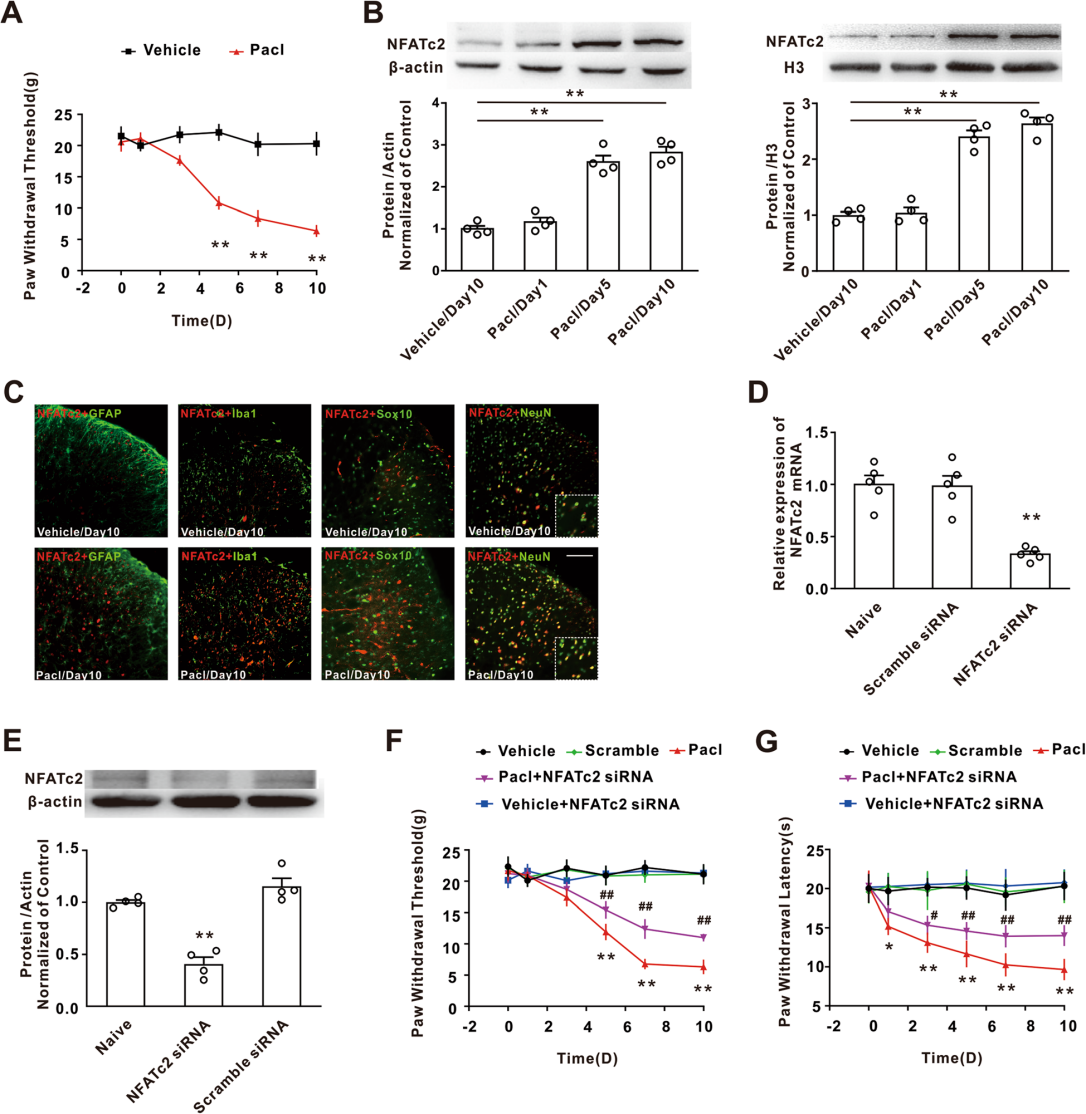
The upregulation of NFATc2 in dorsal horn neurons was involved in the mechanical allodynia induced by paclitaxel. **a** Treatment with paclitaxel decreased the hind paw withdraw threshold in rats (**P < 0.01 vs the vehicle group/day 10, *n* = 12 per group). **b** Representative blots and histogram showed the upregulation of NFATc2 following paclitaxel application on both total and nucleus level (**P < 0.01 vs the vehicle group/day 10, *n* = 4 per group). **c** The immunofluorescence signal of NFATc2 (red) was colocalized with NeuN (neurons marker, green), but not Iba-1 (microglia marker, green) or GFAP (astrocyte marker, green) or Sox10 (oligodendrocyte maker, green) in both vehicle group and paclitaxel group. Left scale bar, 100 μm; right scale bar, 20 μm (*n* = 3 per group). **d**, **e** Intrathecal injection of the NFATc2 siRNA significantly decreased the expression of NFATc2 mRNA and protein (**P < 0.01 vs the scramble group, *n* = 5 or 4 per group). **f**, **g** Deletion of NFATc2 in dorsal horn significantly attenuated the mechanical allodynia and thermal hyperalgesia induced by paclitaxel in the rats (**P < 0.01 vs the vehicle group, ^##^*P* < 0.01 vs the paclitaxel group, *n* = 12 per group)

Intraperitoneal injection of paclitaxel (3 × 8 mg/kg, cumulative dose 24 mg/kg) markedly decreased the mechanical withdrawal threshold compared to the vehicle group (Fig. 1a). Western blotting analysis showed that paclitaxel treatment significantly increased the NFATc2 expression on days 5 and 10, but not day 1, in the spinal dorsal horn on both total and nucleus preparation compared with the vehicle group on day 10 (Fig. 1b). The enhanced NFATc2 was exclusively expressed in the NeuN-positive cells, but not Iba-1, GFAP, or Sox10-positive cells in both paclitaxel and vehicle group (Fig. 1c). To define the role of NFATc2 in paclitaxel-induced mechanical allodynia and thermal hyperalgesia, we synthesized and validated the NFATc2 siRNA. Behavioral test showed that intrathecal injection of the NFATc2 siRNA for consecutive 10 days, which decreased the levels of NFATc2 mRNA and protein in dorsal horn (Fig. 1d, e), significantly attenuated the mechanical allodynia and thermal hyperalgesia induced by paclitaxel (Fig. 1f, g). These results suggested that the spinal NFATc2 was involved in the chronic pain induced by chemotherapeutic paclitaxel.

### Annotation of chromatin immunoprecipitation sequencing of NFATc2 binding sites

**Fig. 2:**
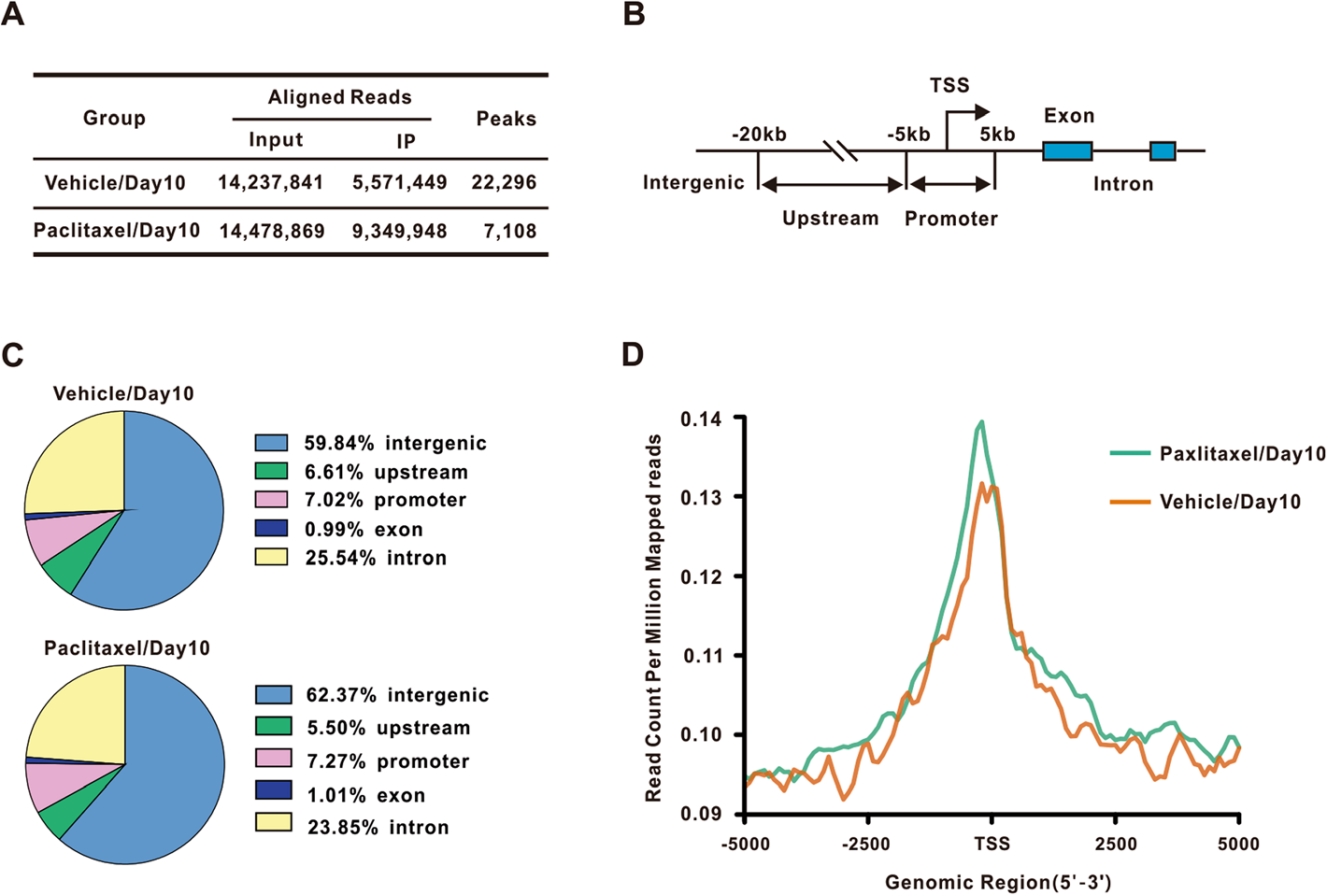
Analysis of chromatin immunoprecipitation sequencing (ChIP-seq) of NFATc2 binding sites. **a** The number of aligned reads and the number of identified peaks in vehicle group/day 10 and paclitaxel group/day 10. **b** Schematic diagram illustrated binding site locations. **c** The genomic distribution (%) of the NFATc2 binding sites in vehicle group/day 10 and paclitaxel group/day 10. **d** Line chart showing the binding intensity of NFATc2 around the TSS of target genes

To explore the mechanism underlying NFATc2-mediated chronic pain, the NFATc2 chromatin immunoprecipitation sequencing (ChIP-seq) study was carried out in the spinal dorsal horn on day 10 following paclitaxel treatment. A total of 22,296 enriched peaks were observed in the vehicle sample which derived at the same time point, while the paclitaxel samples exhibited a total of 7108 peaks (Fig. 2a). Subsequently, the distribution characteristics of the NFATc2 binding sites (peaks) were analyzed according to the described methods (Fig. 2b, c) [28]. 7.02% of NFATc2 binding sites were located within a promoter in the vehicle group, while the NFATc2 binding sites in the promoter is 7.27% in the paclitaxel group (Fig. 2c). The results suggested that paclitaxel treatment changed the distribution of the transcription factor NFATc2 binding sites for the promoter. Studies showed that transcription factors binding closer to the TSS (Transcription Start Site) of a gene have a stronger effect on regulation of gene expression [29, 30]. Then, we compared the read count of peaks within the promoter region between the paclitaxel group and vehicle group, and found that the NFATc2 binding peaks was higher around the TSS (Fig. 2d). Taken together, our data suggested that paclitaxel treatment changed the NFATc2 binding site distribution and had a stronger effect on bound genes.

### Paclitaxel treatment enhanced the NFATc2 binding on the CXCL14 promoter

**Fig. 3:**
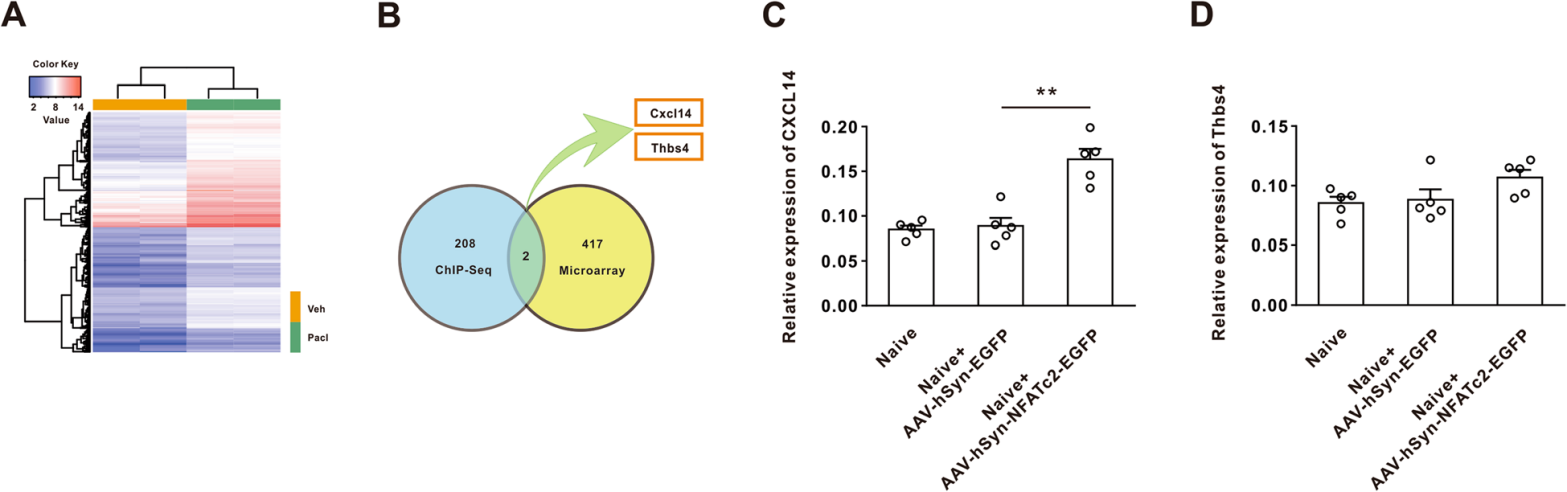
The screening for targeted gene of NAFTc2 binding following paclitaxel treatment. **a** Following the microarray experiment, heat map of 419 genes which increased by 2.5 folds in paclitaxel treatment rats. **b** The intersection of NFATc2 ChIP sequence data with gene expression profile data showed that CXCL14 and Tnbs4 may be regulated by NFATc2 in dorsal horn. **c**, **d** RT-PCR showed CXCL14 but not Thbs4 was significantly increased on day 21 following overexpression of NFATc2 in interspinal AAV-hSyn-NFATc2-EGFP-injected rats (**P < 0.01 vs the AAV-hSyn-EGFP-injected rats, *n* = 5 per group)

It is well-known that NFATc2 is an important inflammation-associated transcription factors, and inflammatory responses play a critical role in chronic pain. To define the proinflammatory cytokine genes for NFATc2 binding site following paclitaxel, we first explored the differential binding peaks between the vehicle group and paclitaxel group by using diffReps analysis. One hundred ninety-three differential binding peaks on the promoter region were observed (Supplementary Table 4), which were mapped to 210 genes. To determine the NFATc2-responsive genes in dorsal horn which were modulated by paclitaxel, we designed a microarray experiment to compare the gene expression profile. Compared with the vehicle group, the expression of 419 genes were upregulated by more than 2.5 folds in paclitaxel rats (Fig. 3a). Next, we integrated the NFATc2 ChIP-seq data with gene expression profile data in order to identify the targets of NFATc2, by which we found that CXCL14 and Thbs4 may be regulated by NFATc2 in dorsal horn (Fig. 3b). RT-PCR results further showed that the expression of CXCL14 mRNA, but not Thbs4, was significantly increased on day 21 following the NFATc2 overexpression in AAV-hSyn-NFATc2-EGFP-injected rats, relative to the AAV-hSyn-EGFP-injected rats (Fig. 3c, d). Taken together, the results suggested that chemokine CXCL14 may be the target gene for transcript factor NFATc2 following paclitaxel treatment.

### CXCL14 upregulation contributed to the mechanical allodynia following paclitaxel treatment

**Fig. 4:**
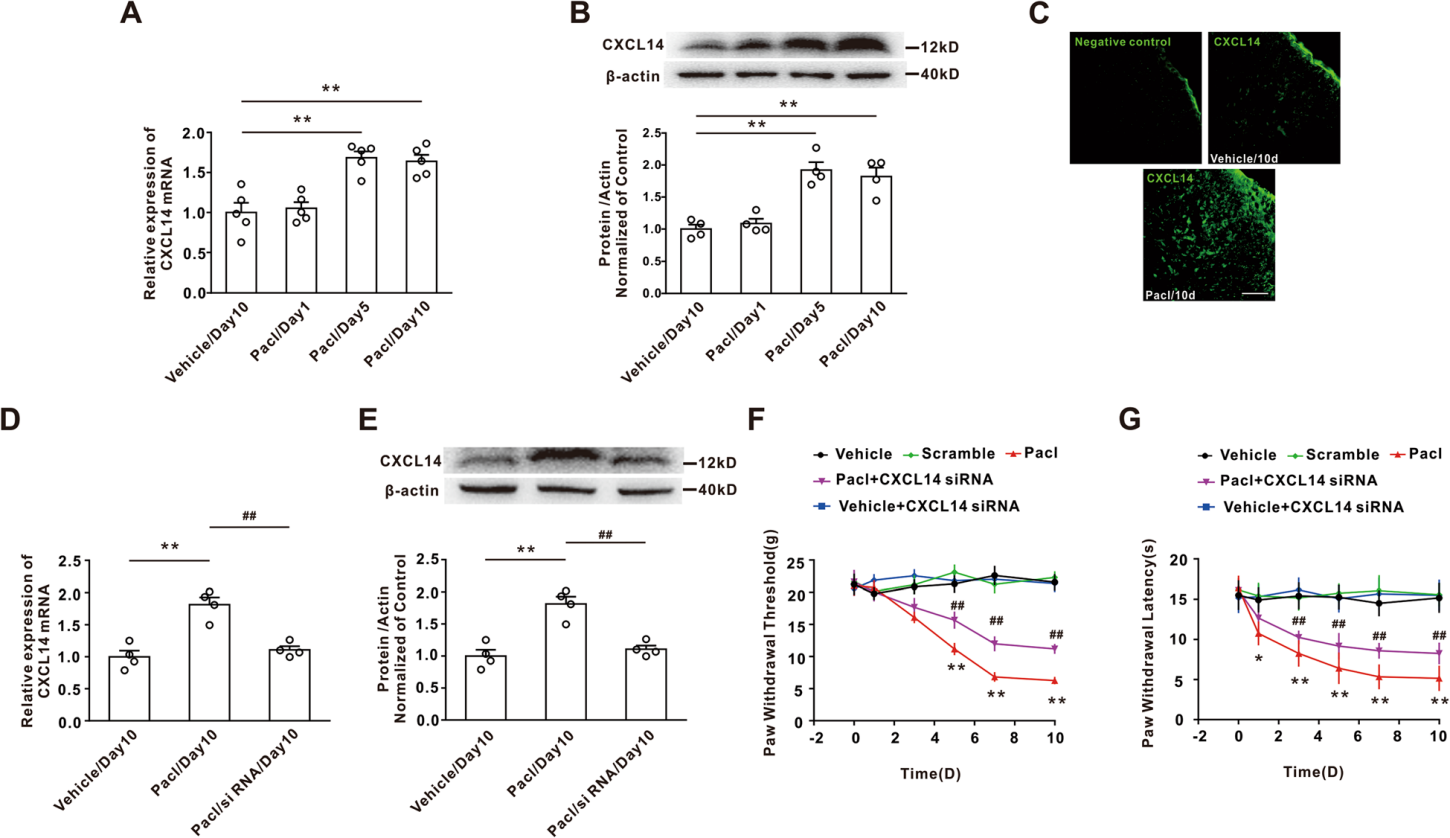
The upregulation of CXCL14 in dorsal horn neurons was involved in mechanical allodynia induced by paclitaxel. **a**, **b** Treatment of paclitaxel significantly increased the expression of CXCL14 mRNA and protein on day 5 and day 10 (**P < 0.01 vs the vehicle group, *n* = 5 per group in real time qPCR, *n* = 4 per group in western blotting). **c** The immunohistochemical staining showed that the paclitaxel treatment increased the CXCL14 expression on day 10 compared with vehicle group. Scale bar, 100 μm (*n* = 3 per group). **d**, **e** Intrathecal injection of CXCL14 siRNA significantly attenuated the upregulation of CXCL14 mRNA and protein expression on day 10 following paclitaxel treatment (**P < 0.01 vs the vehicle group, ^##^*P* < 0.01 vs the paclitaxel treatment, *n* = 5 or 4 per group). **f**, **g** Intrathecal injection of CXCL14 siRNA attenuated both mechanical allodynia and thermal hyperalgesia induced by paclitaxel application (**P < 0.01 vs the vehicle group, ^##^*P* < 0.01 vs the paclitaxel treatment, *n* = 12 per group)

Next, we found that paclitaxel treatment significantly enhanced the expression of CXCL14 mRNA and protein on days 5 and 10 (Fig. 4a, b). Immunohistochemical staining also confirmed that the CXCL14 expression was increased on day 10 after paclitaxel treatment compared with that in the vehicle group (Fig. 4c). To determine whether the upregulation of dorsal horn CXCL14 contributed to mechanical allodynia induced by paclitaxel, intrathecal injection of CXCL14 siRNA for 10 consecutive days was performed. The results showed that application of CXCL14 siRNA, which decreased CXCL14 mRNA and protein expression in dorsal horn (Fig. 4d, e), significantly attenuated the mechanical allodynia and thermal hyperalgesia induced by paclitaxel application (Fig. 4f, g). These results suggested that the upregulation of dorsal horn CXCL14 mediated the chronic pain induced by paclitaxel.

### NFATc2 mediated the upregulation of CXCL14 following paclitaxel treatment

**Fig. 5:**
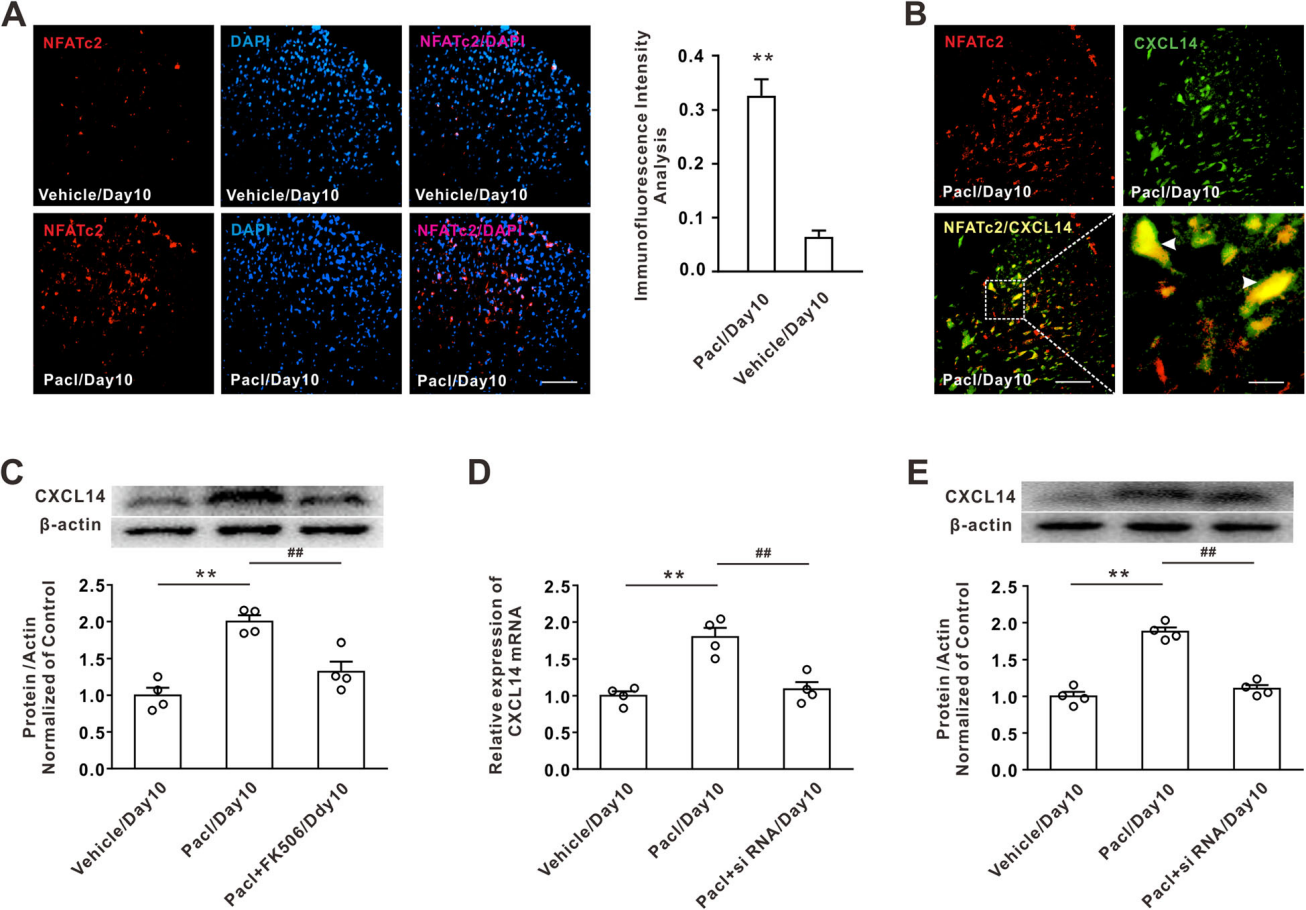
NFATc2 contributed to the CXCL14 upregulation in dorsal horn following paclitaxel treatment. **a** Immunofluorescence staining of NFATc2 showed the intensity of NFATc2 (red) was increased in DAPI (blue) positive nuclear region by paclitaxel application on day 10. The histogram analysis of immunofluorescence intensity demonstrated indicates the increased of nucleus translocation of NFATc2 following paclitaxel treatment. Scale bar, 100 μm (**P < 0.01 vs the vehicle group, *n* = 3 per group). **b** Double immunostaining showed NFATc2 (red) was colocalized with CXCL14 (green). Left scale bar, 100 μm; right scale bar, 20 μm (*n* = 3 per group). **c** Western blotting showed that intrathecal application of FK506 attenuated CXCL14 upregulation induced by paclitaxel (**P < 0.01 vs the vehicle group, ^##^*P* < 0.01 vs the paclitaxel treatment, *n* = 4 per group). **d**, **e** Intrathecal injection of NFATc2 siRNA significantly decreased the increase of CXCL14 mRNA and protein induced by paclitaxel (**P < 0.01 vs the vehicle group, ^##^*P* < 0.01 vs the paclitaxel treatment, *n* = 4 per group)

NFATc2 can translocate to the nucleus to regulate the cytokine and chemokine expression [31]. In this study, we found that paclitaxel treatment significantly increased the intensity of NFATc2 immunofluorescence in the DAPI-positive nuclear region (Fig. 5a). Double immunostaining results showed that the NFATc2 expression was colocalized with CXCL14-positive cells (Fig. 5b). Furthermore, western blotting showed that intrathecal application of NFATc2 inhibitor FK506, which prevented the NFATc2 into the nuclear [32], significantly attenuated the CXCL14 upregulation induced by paclitaxel (Fig. 5c). Finally, we observed that the suppression of NFATc2 in the dorsal horn region produced by intrathecal injection of NFATc2 siRNA significantly decreased the CXCL14 upregulation induced by paclitaxel (Fig. 5d, e). These results suggest that upregulation of CXCL14 after paclitaxel treatment was dependent on the NFATc2 pathway.

### Epigenetic mechanism mediated the CXCL14 upregulation

**Fig. 6:**
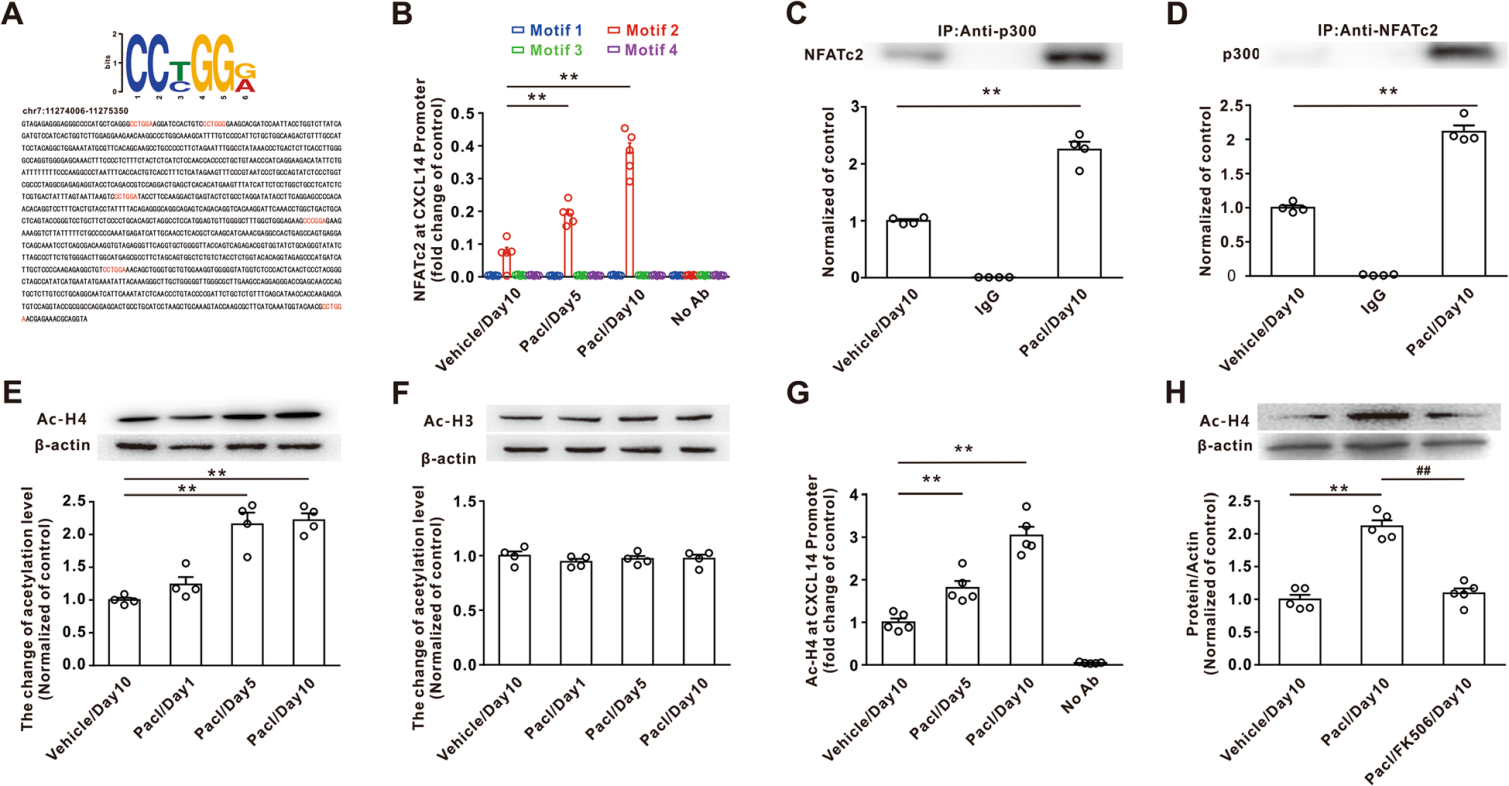
Increased interactions between NFATc2 and p300 increased the level of acetylated histone H4 on the special region area of CXCL14 promoter. **a** Six potential motif for NFATc2 binding on CXCL14 promoter. **b** ChIP-PCR assay was performed with CXCL14 antibody on day 5 and day 10 after paclitaxel treatment in rats using different primer (**P < 0.01 vs vehicle, *n* = 5 per group). **c** NFATc2 was significantly increased in immunocomplex precipitated by p300 antibody on day 10 after paclitaxel treatment (**P < 0.01 vs the vehicle group, *n* = 4 per group). **d** p300 content was increased precipitated by NFATc2 antibody following paclitaxel treatment on day 10 (**P < 0.01 vs the vehicle group, *n* = 4 per group). **e** Western blotting showed that the acetylation of H4 significantly increased on day 5 and day 10 after application of paclitaxel (**P < 0.01 vs the vehicle group, *n* = 4 per group). **f** Application of paclitaxel did not change the acetylation of H3 in dorsal horn (*n* = 4 per group). **g** H4 acetylation on the NFATc2 binding region in CXCL14 promoter was enhanced on day 10 after application of paclitaxel (**P < 0.01 vs the vehicle group, *n* = 5 per group). **h** Intrathecal application of FK506 (NFATc2 inhibitor) decreased the increase of H4 acetylation on CXCL14 promoter on day 10 following paclitaxel application (**P < 0.01 vs the vehicle group, ^##^*P* < 0.01 vs the paclitaxel treatment, *n* = 5 per group)

Chromatin remodeling through the histone acetylation play critical role in inflammatory gene transcription [33]. In the current study, we observed the six motifs for NFATc2 binding on the CXCL14 promoter (Fig. 6a). To determine whether the NFATc2 induced chromatin remodeling to regulate the expression of CXCL14, we designed the four pair primers and examined the binding site of NFATc2 in *CXCL14* promoter in dorsal horn using a ChIP-PCR assay. The DNA, which precipitated by the NFATc2 antibody, was subjected to PCR to amplify different length fragments of the CXCL14 promoter which contained the NFATc2-binding motif with the designed different primers (Supplementary Table 3). The results of qPCR analysis revealed that the binding of NFATc2 in the *CXCL14* promoter region (chr17: 11274394-11274607) in the dorsal horn was significantly enhanced after paclitaxel treatment on days 5 and 10 relative to vehicle group (Fig. 6b).

To assess mechanisms by which paclitaxel regulates the transcription of CXCL14 promoter, immunoprecipitation (IP) was performed on lysates from spinal dorsal horn tissue. The results showed that paclitaxel treatment significantly increased the NFATc2 content on day 10 in the immunocomplex precipitated with p300 antibody when compared with that in vehicle group (Fig. 6c). In addition, the increased p300 content in the immunocomplex precipitated by NFATc2 antibody further confirmed that paclitaxel treatment enhanced the interaction between NFATc2 and p300 in dorsal horn (Fig. 6d). Since p300 played an important role in the modulation of histone acetylation, we further examined whether paclitaxel treatment could modify histone acetylation in the CXCL14 promoter region. Western blot results showed that paclitaxel treatment significantly increased the global acetylation of histone H4, but not H3 (K9) (Fig. 6e, f), on day 10. Next, the DNA was precipitated by the acetylated H4 antibody, and PCR was subsequently performed to amplify the CXCL14 promoter region flanking the NFATc2/p300-binding site. The ChIP assay revealed that the level of H4 acetylation on the CXCL14 promoter motif region was enhanced on day 10 following paclitaxel treatments (Fig. 6g). Importantly, intrathecal injection of FK506 reduced the increases of H4 acetylation induced by paclitaxel treatment (Fig. 6h). These results indicated that paclitaxel treatment induced an increase of histone H4 acetylation at the CXCL14 promoter through interaction between NFATc2 and p300.

## Discussion

In the present study, we provided evidence that transcription factor NFATc2 mediated epigenetic upregulation of chemokine CXCL14 in dorsal horn neurons after paclitaxel application. NFAT, as a key regulator of immune response, was first discovered in T cells as transcriptional activators of interleukin-2 [3]. Currently, the NFAT gene family, including NFATc1-c4, is found to play critical roles in many biological processes in vertebrates. Studies showed that NFAT gene family was correlated with the neuropathic pain following chemotherapeutic oxaliplatin treatment [34]. Furthermore, NFATc4 subtype upregulated the pronociceptive cytokines in DRG and contributed to the chronic pain after nerve injury [13]. In addition, the study showed that NFAT isoforms such as NFATc3 and NFATc4 presented the distinct activation properties in neuron s[35]. Our data first indicated that paclitaxel treatment significantly increased the expression of NFATc2 subtype in dorsal horn neurons, and inhibition of NFATc2 by intrathecal injection of NFATc2 siRNA attenuated the paclitaxel-induced mechanical allodynia. These results suggested that spinal NFATc2 upregulation was involved in the chemotherapeutic paclitaxel-induced chronic pain.

Evidence showed that NFATc2 directly regulated a wide range of chemokine genes with broad biological effects [28]. Here, we conducted genome-wide mapping of NFATc2 binding sites with or without paclitaxel treatment, and found that paclitaxel treatment shifted the distribution of NFATc2 binding sites much closer to the TTS. Furthermore, we compared the differential binding peaks between the paclitaxel group and the vehicle group and found that 193 differential binding peaks were mapped to 210 genes. Indeed, NFAT regulated the chemokine expression in the CNS. For example, it was previously reported that the expression of CXCL2 was regulated by NFAT in microglia [36]. In the present study, by integrating ChIP-sequence data with gene expression profile, we identified CXCL14 as the target gene of transcription factor to mediate chronic pain induced by paclitaxel. It was consistent with previous reports that NFATc2 can bind to several chemokine genes or receptors, including CXCL14 or CCR2 [28, 37]. We further found that NFATc2 inhibitor FK506 prevented the upregulation of chemokine CXCL14 induced by paclitaxel, which was in line with the previous finding that inhibition of NFAT signaling decreased the cytokine production during fungal infection [38].

CXCL14 was initially identified from breast and kidney cells and was widely expressed in normal tissue [39]. In the present study, paclitaxel treatment increased the expression of CXCL14 mRNA in spinal dorsal horn, suggested that the transcript process was involved in the CXCL14 upregulation. Furthermore, deletion of CXCL14 significantly ameliorated the mechanical allodynia induced by paclitaxel. Although the identification of receptor for CXCL14 still remains obscure, evidence showed that CXC chemokine receptor CXCR4 and CXCR7, as G protein-coupled cell-surface receptor, have high affinity for CXCL14 [40, 41]. CXCL14, as an emerging immune and inflammatory chemokine, induced intracellular signaling through G protein-coupled cell-surface receptor and increased the neuronal excitability [42], which contributed to the paclitaxel-induced mechanical allodynia. Chromatin remodeling by epigenetic modification such as histone acetylation contributed to the gene expression. It has been reported that the proximity of transcription factor binding sites to the TSS of a given gene can predict transcriptional activity [43]. In the present study, we found that paclitaxel treatment altered the distribution of NFATc2 binding site, within the loci much closer to the TSS of target genes, which suggested the enhanced transcriptional activity of NFATc2 after paclitaxel treatment. In addition, we found that paclitaxel treatment enhanced the interaction between NFATc2 and p300, and NFATc2 binding sites motif on CXCL14 gene was adjacent to the predicated p300 binding site motif, which suggested that the histone acetylation may be involved in the CXCL14 upregulation induced by paclitaxel. Indeed, H4 acetylation on the CXCL14 promoter is significantly increased following paclitaxel treatment. Studies showed that activation of NFATc1/2 critically regulated the expression of several key cytokines including IL-2. In the present study, we found that intrathecal injection of NFATc2 inhibitor FK506, which prevented the NFATc2 translocation into the nucleus, significantly decreased the CXCL14 upregulation induced by paclitaxel. These results suggested that the enhanced nuclear translocation of NFATc2 promoted the histone H4 acetylation and contributed to the CXCL14 upregulation following paclitaxel treatment.

## Conclusions

Altogether, our study showed that application of paclitaxel induced NFATc2 transcriptional activity, mediated epigenetic upregulation of CXCL14 in spinal dorsal horn, and contributed to mechanical allodynia. This provided a novel potential target for the treatment of chemotherapeutic-induced neuropathic pain.

## Supplementary information

Additional file 1: ** Figure S1.** (PDF 138 kb)

Additional file 2: **Table S1.** The Nucleotide Sequences of CXCL14 or NFATc2. (DOC 14 kb)

Additional file 3: **Table S2.** The specific primer sequences. (DOC 2 kb)

Additional file 4: **Table S3.** The examined sequence and primer. (DOC 1 kb)

Additional file 5: **Table S4.** Differentially enriched peaks in promoter region. (DOC 42 kb)

## Data Availability

The data used and/or analyzed during the current study are available from the corresponding author on reasonable request.

## Abbreviations

NFATc2: Nuclear factor of activated T cells 2
CXCL14: C-X-C motif chemokine ligand 14
CXCL12: C-X-C motif chemokine ligand 12
CX3CL1: C-X3-C motif chemokine ligand 1
CCL2: C-C motif chemokine ligand 2
CNS: Central nervous system
DRG: Dorsal root ganglion
mRNA: Message RNA
siRNA: Small interfering RNA
TSS: Transcription start site
H3: Histone 3
H4: Histone 4
ChIP-seq: Chromatin immunoprecipitation sequence
Co-IP: Coimmunoprecipitation
