# Supplementary figures and images for: NFATc2-dependent epigenetic upregulation of CXCL14 is involved in the development of neuropathic pain induced by paclitaxel
Source: J Neuroinflammation. 2020 Oct 18;17:310. doi: 10.1186/s12974-020-01992-1 (PMC7570122; doi:10.1186/s12974-020-01992-1)

Supplementary Figure.1

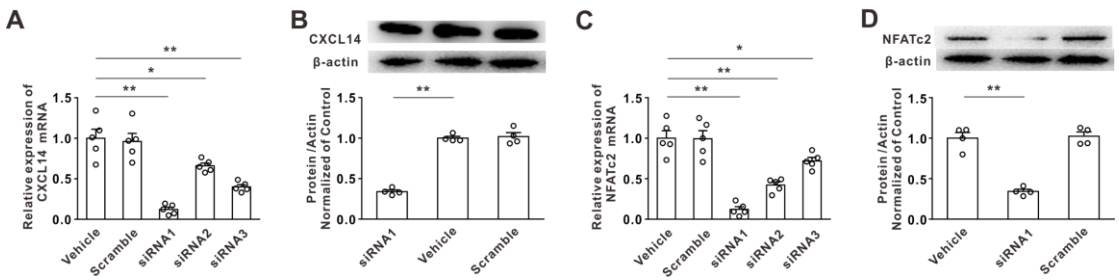

Supplement: Supplementary file 1 — Figure S1. (PDF 138 kb) [file 12974_2020_1992_MOESM1_ESM.pdf]
