# Supplementary material for: NFATc2-dependent epigenetic upregulation of CXCL14 is involved in the development of neuropathic pain induced by paclitaxel
Source: J Neuroinflammation. 2020 Oct 18;17:310. doi: 10.1186/s12974-020-01992-1 (PMC7570122; doi:10.1186/s12974-020-01992-1)
Supplement: Supplementary file 2 — Table S1. The Nucleotide Sequences of CXCL14 or NFATc2. (DOC 14 kb) [file 12974_2020_1992_MOESM2_ESM.doc]

**Table 1.** The Nucleotide Sequences of CXCL14 or NFATc2

| GENE Name SEQUENCE |
| --- |
| CXCL14 siRNA1 5’- UGAAGAAGCUGGAAAUGAA dTdT-3’ (sense)  3’-dTdTACUUCUUCGACCUUUACUU-5’ (antisense)  siRNA2 5’- GGUUUCAUAUUCUUUCUAA dTdT-3’ (sense)  3’-dTdT UUAGAAAGAAUAUGAAACC-5’ (antisense)  siRNA3 5’- CACAAAUUAUAUAUUGUUA dTdT-3’ (sense)  3’-dTdT UAACAAUAUAUAAUUUGUG-5’ (antisense)  NFATc2 siRNA1 5’-CCGUGAAAGUGAACUUCUA dTdT-3’ (sense)  3’-dTdT UAGAAGUUCACUUUCACGG-5’ (antisense)  siRNA2 5’- CCUUCUCAGCCUUAAGUUA dTdT-3’ (sense)  3’-dTdT UAACUUAAGGCUGAGAAGG-5’ (antisense)  siRNA3 5’- ACGGCUACAUGGAGAACAA dTdT-3’ (sense)  3’-dTdT UUGUUCUCCAUGUAGCCGU-5’ (antisense) |
