# Supplementary material for: NFATc2-dependent epigenetic upregulation of CXCL14 is involved in the development of neuropathic pain induced by paclitaxel
Source: J Neuroinflammation. 2020 Oct 18;17:310. doi: 10.1186/s12974-020-01992-1 (PMC7570122; doi:10.1186/s12974-020-01992-1)
Supplement: Supplementary file 3 — Table S2. The specific primer sequences. (DOC 2 kb) [file 12974_2020_1992_MOESM3_ESM.doc]

**Table 2.** The specific primer sequences

| GENE Primer Sequence |
| --- |
| NFATc2 Forward 5’- CTACCCCACGGTCATCCAAC -3’  Reverse 3’-CCTTCCTGATGATTTCATTAACGTC-5’  CXCL14 Forward 5’- GACGGGTCCAAGTGTAAGTGTTCC -3’  Reverse 3’- TCTTCTCCTCGCAGTGTGGGTAC -5’  Thbs4 Forward 5’- GACTCCTGTGACACCAACCAAGAC -3’  Reverse 3’- TCATCGCATTCATCGCCAATCCC -5’ |
