# Supplementary material for: NFATc2-dependent epigenetic upregulation of CXCL14 is involved in the development of neuropathic pain induced by paclitaxel
Source: J Neuroinflammation. 2020 Oct 18;17:310. doi: 10.1186/s12974-020-01992-1 (PMC7570122; doi:10.1186/s12974-020-01992-1)
Supplement: Supplementary file 4 — Table S3. The examined sequence and primer. (DOC 1 kb) [file 12974_2020_1992_MOESM4_ESM.doc]

**Table 3.** The examined sequence and primer

| The checked sequence 1 (Length: 327bp, chr17:11274006-11274332)  GTAGAGAGGGAGGGCCCCATGCTCAGGGCCTGGAAGGATCCACTGTCCCTGGGGAAGCACGATCCAATTACCTGGTCTTATCAGATGTCCATCACTGGTCTTGGAGGAAGAACAAGGCCCTGGCAAAGCATTTTGTCCCCATTCTGCTGGCAAGACTGTTTGCCATTCCTACAGGCTGGAAATATGCGTTCACAGCAAGCCTGCCCCCTTCTAGAATTTGGCCTATAAACCCTGACTCTTCACCTTGGGGCCAGGTGGGGAGCAAACTTTCCCCTCTTTCTACTCTCATCTCCAACCACCCCTGCTGTAACCCATCAGGAAGACATA  ***Primer：****F：**GTAGAGAGGGAGGGCCCCATG*  *R：**TATGTCTTCCTGATGGGTTACAGC* |
| --- |
| The checked sequence 2 (Length: 214bp,chr17:11274394-11274607)  CGTAATCCCTGCCAGTATCTCCCTGGTCGCCCTAGGCGAGAGAGGTACCTCAGACCGTCCAGGACTGAGCTCACACATGAAGTTTATCATTCTCCTGGCTGCCTCATCTCTCGTGACTATTTAGTAATTAAGTCCCTGGATACCTTCCAAGGACTGAGTACTCTGCCTAGGATATACCTTCAGGAGCCCCACAACACAGGTCCTTTCACTGTAC  ***Primer：****F：**CGTAATCCCTGCCAGTATCTC*  *R：**GTACAGTGAAAGGACCTGTGT* |
| The checked sequence 3 (Length: 273bp,chr17:11274684-11274956)  CTGCTTCTCCCCTGCACAGCTAGGCCTCCATGGAGTGTTGGGGCTTTGGCTGGGAGAAGCCCGGAGAAGAAAGGTCTTATTTTTCTGCCCCCAAATGAGATCATTGCAACCTCACGCTCAAGCATCAAACGAGGCCACTGAGCCAGTGAGGATCAGCAAATCCTCAGCGACAAGGTGTAGAGGGTTCAGGTGCTGGGGTTACCAGTCAGAGACGGTGGTATCTGCAGGGTATATCTTAGCCCTTCTGTGGGACTTGGCATGAGCGCCTTCTAG  ***Primer：****F：CTGCTTCTCCCCTGCACAGCT*  *R：CTAGAAGGCGCTCATGCCAAGT* |
| The checked sequence 4 (Length: 351bp,chr17:11275000-11275350)  CATTGCTCCCCAAGAGAGGCTGTCCTGGAAACAGCTGGGTGCTGTGGAAGGTGGGGGTATGGTCTCCCACTCAACTCCCTACGGGCTAGCCATATCATGAATATGAAATATTACAAAGGGCTTGCTGGGGGTTGGGCGCTTGAAGCCAGGAGGGACCGAGCAACCCAGTGCTCTTGTCCTGCAGGCAATCATTCAAATATCTCAACCCTGTACCCCGATTCTGCTCTGTTTCAGCATAACCACCAAGAGCATGTCCAGGTACCGCGGCCAGGAGCACTGCCTGCATCCTAAGCTGCAAAGTACCAAGCGCTTCATCAAATGGTACAACGCCTGGAACGAGAAACGCAGGTA  ***Primer：****F：CATTGCTCCCCAAGAGAGGCT*  *R：TACCTGCGTTTCTCGTTCCAG* |
