# Supplementary material for: NFATc2-dependent epigenetic upregulation of CXCL14 is involved in the development of neuropathic pain induced by paclitaxel
Source: J Neuroinflammation. 2020 Oct 18;17:310. doi: 10.1186/s12974-020-01992-1 (PMC7570122; doi:10.1186/s12974-020-01992-1)
Supplement: Supplementary file 5 — Table S4. Differentially enriched peaks in promoter region. (DOC 42 kb) [file 12974_2020_1992_MOESM5_ESM.doc]

**Supplementary Table 4 Differentially enriched peaks in promoter region**

| **Peak region** | **Transcript** | **Gene symbol** | **FDR** |
| --- | --- | --- | --- |
| chr1:81877701-81880700 | NM_138828 | Apoe | 0 |
| chr1:81877701-81880700 | NM_001270682 | Apoe | 0 |
| chr1:81877701-81880700 | NM_001270681 | Apoe | 0 |
| chr1:81877701-81880700 | NM_001270684 | Apoe | 0 |
| chr1:81877701-81880700 | NM_012824 | Apoc1 | 0 |
| chr1:81877701-81880700 | NM_001270683 | Apoe | 0 |
| chr1:81877701-81880700 | NM_001109996 | Apoc1 | 0 |
| chr18:1184101-1190900 | NM_001047850 | Thoc1 | 3.244E-07 |
| chr1:260072901-260079200 | NM_012732 | Lipa | 5.367E-07 |
| chr2:135057101-135062500 | NM_001108548 | Rpl22l1 | 1.166E-06 |
| chr1:203869401-203874100 | NM_001039611 | Nsmce1 | 1.426E-06 |
| chr18:30761701-30764500 | NM_001107393 | Diaph1 | 1.763E-06 |
| chr8:39271901-39276500 | NM_001025660 | Ei24 | 1.892E-06 |
| chr8:56698701-56701500 | NM_001108147 | Npat | 4.81E-06 |
| chr8:56698701-56701500 | NM_001106821 | Atm | 4.81E-06 |
| chr7:141473901-141479200 | NM_001008324 | Eif4b | 7.67E-06 |
| chr8:95579601-95584900 | NM_001108174 | Snx14 | 1.204E-05 |
| chr19:64454101-64458300 | NM_001107436 | RGD1304884 | 1.834E-05 |
| chr6:85204901-85207400 | NM_001108711 | Snx6 | 2E-05 |
| chr9:29159101-29162700 | NM_001134597 | Fam135a | 2E-05 |
| chr7:137481001-137486200 | NM_001271170 | Scaf11 | 2.687E-05 |
| chr4:224128401-224132700 | NM_001002804 | C1rl | 3.045E-05 |
| chr15:65488801-65492000 | NM_053520 | Elf1 | 3.045E-05 |
| chr8:119785101-119790800 | NM_001135045 | Arpp21 | 3.679E-05 |
| chr8:119785101-119790800 | NM_001135046 | Arpp21 | 3.679E-05 |
| chr1:198725301-198729700 | NM_001100519 | Gga2 | 3.679E-05 |
| chr13:78129301-78132600 | NM_001011906 | Acbd6 | 3.98E-05 |
| chr7:70538401-70542000 | NM_023026 | Agap2 | 4.588E-05 |
| chr10:36483301-36488700 | NM_001017472 | LOC497899 | 5.113E-05 |
| chr10:36483301-36488700 | NM_001013041 | Clk4 | 5.113E-05 |
| chr18:56338601-56343100 | NM_001106142 | Afap1l1 | 5.788E-05 |
| chrX:13904401-13906700 | NM_206950 | Mid1ip1 | 6.895E-05 |
| chr3:43838301-43840800 | NM_001024310 | Arl6ip6 | 6.895E-05 |
| chr3:43838301-43840800 | NM_001106480 | Prpf40a | 6.895E-05 |
| chr17:47771101-47773500 | NM_031093 | Rala | 6.895E-05 |
| chr1:283353701-283356800 | NM_001106200 | Tectb | 6.895E-05 |
| chr16:47834801-47838100 | NM_001134863 | Stox2 | 6.895E-05 |
| chr5:66146101-66148400 | NM_013044 | Tmod1 | 6.895E-05 |
| chr4:28603001-28606000 | NM_001034015 | Calcr | 6.895E-05 |
| chr4:28603001-28606000 | NM_053816 | Calcr | 6.895E-05 |
| chr7:74278601-74283700 | NM_001134886 | Vps13b | 8.227E-05 |
| chr18:31104501-31109800 | NM_053944 | Pcdh12 | 8.227E-05 |
| chr18:15907301-15911800 | NM_024373 | Galnt1 | 8.227E-05 |
| chr3:171099001-171101400 | NM_001347532 | Adnp | 0.0001027 |
| chr16:62142501-62145300 | NM_001191554 | Purg | 0.0001027 |
| chr17:81941001-81943600 | NM_001109403 | C1ql3 | 0.0001027 |
| chr15:63762601-63765000 | NM_001044257 | Epsti1 | 0.0001027 |
| chr11:84850401-84852700 | NM_001012016 | Tbccd1 | 0.0001027 |
| chr19:26246701-26249400 | NM_001047967 | LOC501233 | 0.0001027 |
| chr19:26246701-26249400 | NM_031565 | Ces1e | 0.0001027 |
| chr1:175606101-175609100 | NM_001014023 | Trim5 | 0.0001027 |
| chr1:206986501-206989200 | NM_001011936 | Bag3 | 0.0001027 |
| chr9:22958701-22962900 | NM_001012130 | Pgk2 | 0.0001141 |
| chr10:109334801-109338300 | NM_012998 | P4hb | 0.0001396 |
| chr18:71672101-71676000 | NM_001277450 | Smad2 | 0.0001396 |
| chr18:71672101-71676000 | NM_019191 | Smad2 | 0.0001396 |
| chr4:30459901-30463800 | NM_053551 | Pdk4 | 0.0001396 |
| chr1:88131201-88134600 | NM_030867 | Nfkbib | 0.0001523 |
| chr1:88131201-88134600 | NM_001008368 | Sirt2 | 0.0001523 |
| chr3:87626501-87629700 | NM_001005562 | Creb3l1 | 0.0001523 |
| chr7:102870401-102873200 | NM_022258 | A1bg | 0.0001523 |
| chr10:84755701-84758700 | NM_001012142 | Scrn2 | 0.0001523 |
| chr16:82473101-82476000 | NM_001017508 | Tex29 | 0.0001523 |
| chr18:69389101-69392700 | NM_001011924 | Mbd1 | 0.0001559 |
| chr5:2870601-2874500 | NM_001012464 | Terf1 | 0.0001606 |
| chr1:86313201-86317300 | NM_001025643 | Fbl | 0.000195 |
| chr19:69096501-69099000 | NM_001009704 | Sipa1l2 | 0.000195 |
| chr17:21607801-21610200 | NM_057188 | Gmpr | 0.0002163 |
| chr4:232658801-232661500 | NM_001109603 | Mansc1 | 0.0002163 |
| chr6:136071401-136073800 | NM_001271253 | Unc79 | 0.0002163 |
| chr3:161559101-161561900 | NM_133582 | Blcap | 0.0002163 |
| chr3:161559101-161561900 | NM_053601 | Nnat | 0.0002163 |
| chr3:161559101-161561900 | NR_073089 | Nnat | 0.0002163 |
| chr3:161559101-161561900 | NM_001270867 | Nnat | 0.0002163 |
| chr3:161559101-161561900 | NM_181687 | Nnat | 0.0002163 |
| chr8:36775501-36778000 | NM_031630 | Ddx25 | 0.0002163 |
| chr1:23094201-23097300 | NM_019370 | Enpp3 | 0.0002163 |
| chr10:33895301-33897400 | NR_144448 | LOC100360106 | 0.0002163 |
| chr7:27343801-27346600 | NM_001135566 | LOC691921 | 0.0002781 |
| chr4:136452701-136455700 | NM_022522 | Casp2 | 0.0002781 |
| chr18:50877801-50880900 | NM_001109126 | Tex43 | 0.0002781 |
| chr10:29949701-29956000 | NM_001014117 | Ublcp1 | 0.0002912 |
| chr7:135458201-135463500 | NM_001106791 | Irak4 | 0.000292 |
| chr7:54777401-54781700 | NM_001108094 | Krr1 | 0.000292 |
| chr3:26127101-26132100 | NM_020106 | Olr414 | 0.0003089 |
| chr9:79885801-79890100 | NM_013122 | Igfbp2 | 0.0003089 |
| chr4:232673701-232676400 | NM_001108650 | Borcs5 | 0.0003097 |
| chr20:24968901-24971000 | NM_001191719 | Jmjd1c | 0.0003097 |
| chr8:97030401-97033500 | NM_001047101 | Adamts7 | 0.0003097 |
| chr9:84328401-84331000 | NM_057107 | Acsl3 | 0.0003097 |
| chr18:22693101-22695800 | NM_022958 | Pik3c3 | 0.0003097 |
| chr19:36195601-36198200 | NM_001107162 | Dcaf15 | 0.0003097 |
| chr4:196089101-196091700 | NM_001034131 | Foxp1 | 0.0003097 |
| chr7:100527701-100530300 | NR_131067 | LOC690120 | 0.0003097 |
| chr14:87293801-87296200 | NM_001103362 | Nudcd3 | 0.0003097 |
| chr9:60815201-60818900 | NM_201990 | Pgap1 | 0.0003788 |
| chr1:204865501-204868800 | NM_134456 | Sh2b1 | 0.0003788 |
| chr1:204865501-204868800 | NM_001048180 | Sh2b1 | 0.0003788 |
| chr3:99048101-99051200 | NM_001107755 | Pamr1 | 0.0003788 |
| chr1:175453901-175456700 | NM_001000165 | Olr148 | 0.0003788 |
| chr4:34091701-34095700 | NM_001173554 | Mios | 0.0003788 |
| chr16:75460301-75464500 | NM_001134744 | Agpat5 | 0.0003788 |
| chr1:171877801-171882100 | NM_172033 | Plekhb1 | 0.0004185 |
| chr5:64011701-64014400 | NM_031974 | Clta | 0.0004432 |
| chr13:65484601-65487200 | NM_001024769 | Cdc73 | 0.0004432 |
| chr1:216691901-216694800 | NM_032614 | Glrx3 | 0.0004432 |
| chrX:124847101-124849800 | NM_017068 | Lamp2 | 0.0004432 |
| chr4:38090001-38092300 | NM_001110492 | Phf14 | 0.0004432 |
| chr12:50042601-50045500 | NM_001126273 | Alkbh2 | 0.0004432 |
| chr10:31305201-31308200 | NM_001108825 | Itk | 0.0004432 |
| chr11:84839901-84842000 | NM_001015021 | Dnajb11 | 0.0004432 |
| chr5:60972201-60974300 | NM_017321 | Aco1 | 0.0004432 |
| chr5:175725701-175728500 | NM_001107997 | Mmel1 | 0.0004432 |
| chr13:54486201-54488800 | NM_001160313 | Nfasc | 0.0004432 |
| chr3:130219201-130221200 | NM_001039024 | Spef1 | 0.0004432 |
| chr4:233023601-233026200 | NM_001003403 | Apold1 | 0.0004432 |
| chr7:123078801-123081100 | NM_024398 | Aco2 | 0.0004432 |
| chr7:123078801-123081100 | NM_138888 | Phf5a | 0.0004432 |
| chr2:41587201-41592000 | NM_017133 | Thbs4 | 0.0004432 |
| chr7:99347601-99350400 | NM_001122976 | Trmt12 | 0.0004432 |
| chr3:125929201-125932400 | NM_001107770 | Sppl2a | 0.0004432 |
| chr1:163891101-163898100 | NM_001015012 | Rab30 | 0.0004432 |
| chr13:31961701-31967700 | NM_001108342 | Kdsr | 0.0004861 |
| chr9:72122301-72126400 | NM_001108221 | Mdh1b | 0.0004872 |
| chr9:72122301-72126400 | NM_001009673 | Fastkd2 | 0.0004872 |
| chr16:11862201-11865800 | NM_001080149 | Ptpn20 | 0.0005258 |
| chr20:426301-429800 | NM_001000894 | Olr1675 | 0.0005258 |
| chr1:263577501-263580900 | NM_024385 | Hhex | 0.0005258 |
| chr2:161914301-161917800 | NM_001004262 | Cog6 | 0.0005258 |
| chr2:115777301-115780700 | NM_001329883 | LOC100360846 | 0.0005258 |
| chr2:133691801-133694900 | NM_001106422 | Tnik | 0.0005258 |
| chr7:139132301-139135700 | NR_036618 | LOC680590 | 0.0005258 |
| chr3:99606801-99609900 | NM_001106492 | Apip | 0.0005258 |
| chr3:10918301-10921400 | NM_001106561 | Cacfd1 | 0.0005515 |
| chr7:124277201-124280500 | NM_130740 | Pacsin2 | 0.0006086 |
| chr5:160327201-160330300 | NM_199108 | Hp1bp3 | 0.0006086 |
| chr10:15853501-15858300 | NM_001108270 | RGD1311343 | 0.0006086 |
| chr5:156011201-156013500 | NM_053983 | Cd52 | 0.0006353 |
| chr2:147378201-147383500 | NM_001014251 | LOC365778 | 0.0006361 |
| chr2:147378201-147383500 | NM_001047910 | Mfsd8 | 0.0006361 |
| chr4:166144701-166147600 | NM_001109242 | RGD1562515 | 0.0006406 |
| chr6:21276701-21279000 | NM_138541 | Epcam | 0.0006406 |
| chr3:124512001-124514400 | NM_001191065 | Shc4 | 0.0006406 |
| chr14:65630701-65633000 | NM_181365 | Kcnip4 | 0.0006406 |
| chr2:212202301-212204400 | NM_021864 | Sprr1a | 0.0006406 |
| chr7:142650001-142652000 | NM_017248 | Hnrnpa1 | 0.0006406 |
| chr14:112805401-112807400 | NR_031929 | Mir217 | 0.0006406 |
| chr14:112805401-112807400 | NR_031928 | Mir216a | 0.0006406 |
| chr18:59592001-59594100 | NM_001008300 | Nedd4l | 0.0006406 |
| chr1:79158801-79161700 | NM_001106226 | Bicra | 0.0006406 |
| chr17:91590701-91592600 | NM_001077635 | Acbd5 | 0.0006406 |
| chr3:38796501-38798500 | NM_001108581 | Epc2 | 0.0006406 |
| chr7:44793301-44795800 | NM_012921 | Alx1 | 0.0006406 |
| chr4:184186701-184188800 | NM_001106608 | Chchd6 | 0.0006406 |
| chr4:136538201-136542500 | NM_053761 | Zyx | 0.0006406 |
| chr3:55266501-55270900 | NM_001109199 | Ifih1 | 0.0006406 |
| chr15:44355601-44360900 | NM_139091 | Nup58 | 0.0006406 |
| chr14:62711701-62715000 | NM_001107215 | Guf1 | 0.0007229 |
| chr19:48985801-48988600 | NM_001107423 | Esrp2 | 0.0007229 |
| chr3:117464301-117466500 | NM_001109204 | Rad51 | 0.0007229 |
| chr3:19247001-19250300 | NM_001106569 | Psmd5 | 0.0007229 |
| chr7:124184901-124187300 | NM_001044273 | Arfgap3 | 0.0007229 |
| chr10:84434301-84437600 | NM_001199169 | Snx11 | 0.0007229 |
| chr10:84434301-84437600 | NM_001012012 | Snx11 | 0.0007229 |
| chr13:81112901-81115600 | NR_032299 | Mir488 | 0.0007229 |
| chr6:36209701-36211900 | NM_053792 | Ift172 | 0.0007229 |
| chr13:83415001-83417700 | NM_001107190 | Rabgap1l | 0.0007229 |
| chr5:139458801-139461600 | NM_001106680 | Toe1 | 0.0007229 |
| chr5:139458801-139461600 | NM_133316 | Mutyh | 0.0007229 |
| chr14:38967201-38970500 | NM_080587 | Gabra4 | 0.0007229 |
| chr6:117510201-117513600 | NM_001108042 | Zfp410 | 0.0007229 |
| chr5:54929101-54936000 | NM_001008879 | Zfp292 | 0.0007609 |
| chr19:36156301-36160700 | NM_001105944 | Rfx1 | 0.0007909 |
| chr7:121302601-121306200 | NM_019166 | Syngr1 | 0.0008127 |
| chr9:53227001-53231000 | NM_001012131 | Inpp1 | 0.0008388 |
| chr8:127987801-127992400 | NM_001030032 | Slc25a38 | 0.0008388 |
| chr19:60245801-60250800 | NM_001008366 | Cenpn | 0.0008945 |
| chr1:235909801-235914000 | NM_001047855 | Dtx4 | 0.0008945 |
| chr14:85015501-85017600 | NM_001006960 | Mtfp1 | 0.0009068 |
| chr1:191241801-191243900 | NM_138513 | Calcb | 0.0009068 |
| chr2:204984701-204986900 | NM_001108555 | Lrba | 0.0009068 |
| chr3:150862101-150864500 | NM_001037350 | LOC296235 | 0.0009068 |
| chr17:11273401-11275900 | NM_001013137 | Cxcl14 | 0.0009068 |
| chr4:65873001-65875100 | NM_173045 | Zc3hav1 | 0.0009068 |
| chr11:34047001-34049000 | NM_001037347 | Scaf4 | 0.0009068 |
| chr19:36290701-36292600 | NR_031825 | Mir23a | 0.0009068 |
| chr19:36290701-36292600 | NR_031828 | Mir24-2 | 0.0009068 |
| chr19:36290701-36292600 | NR_031833 | Mir27a | 0.0009068 |
| chr7:37142601-37144500 | NM_001108086 | Eea1 | 0.0009068 |
| chr7:117662201-117664400 | NM_001130571 | Cpsf1 | 0.0009068 |
| chr7:117662201-117664400 | NM_001077669 | Slc39a4 | 0.0009068 |
| chr2:142874001-142876000 | NM_001025406 | Exosc9 | 0.0009068 |
| chr10:71298101-71300700 | NM_001270836 | Dusp14 | 0.0009068 |
| chr10:71298101-71300700 | NM_001079893 | Dusp14 | 0.0009068 |
| chr10:71298101-71300700 | NM_001270835 | Dusp14 | 0.0009068 |
| chr10:90820901-90822800 | NM_001108838 | C1ql1 | 0.0009068 |
| chr3:2453401-2455400 | NM_001107818 | Ndor1 | 0.0009068 |
| chr3:2453401-2455400 | NM_001107819 | Tmem203 | 0.0009068 |
| chr18:1258001-1260100 | NM_001008301 | Usp14 | 0.0009068 |
| chr3:50652701-50654600 | NM_001108582 | Dapl1 | 0.0009068 |
| chr4:222673201-222675600 | NM_001109352 | Foxj2 | 0.0009068 |
| chr16:10983501-10985900 | NM_001134605 | RGD1561145 | 0.0009068 |
| chr8:61802001-61804200 | NM_001000473 | Olr1325 | 0.0009068 |
| chr2:185756401-185758600 | NM_001107682 | Nmd3 | 0.0009068 |
| chr1:37509501-37511700 | NM_001106097 | Med10 | 0.0009068 |
| chr1:235212601-235214700 | NM_001009647 | Mrpl16 | 0.0009068 |
| chr1:226222801-226224700 | NM_001008369 | Pitpnm1 | 0.0009068 |
| chr13:96686001-96689300 | NM_001000083 | Olr1589 | 0.0009068 |
| chr14:37183001-37185000 | NM_001191677 | Cwh43 | 0.0009068 |
| chr16:14252501-14254500 | NM_053572 | Cdhr1 | 0.0009068 |
| chr18:59031701-59037400 | NM_001106141 | RGD1562699 | 0.0009068 |
| chr15:31687801-31690300 | NM_001014233 | Tmem55b | 0.0009569 |
| chr7:92027001-92030100 | NM_001126266 | Utp23 | 0.0009569 |
| chr10:75667101-75671100 | NM_001108288 | Trim37 | 0.0009569 |
| chr17:32613301-32616600 | NM_001011923 | Prpf4b | 0.0009569 |
| chr5:149025701-149028700 | NM_001276304 | Dlgap3 | 0.0009739 |
| chr5:149025701-149028700 | NM_001301876 | Dlgap3 | 0.0009739 |
| chr5:149025701-149028700 | NM_173138 | Dlgap3 | 0.0009739 |
| chr4:61437601-61440800 | NM_012498 | Akr1b1 | 0.0009739 |
| chr10:56345101-56348200 | NM_001004272 | Phf23 | 0.0009739 |
| chr10:56345101-56348200 | NM_172036 | Gabarap | 0.0009739 |
| chr8:22019101-22021400 | NM_053354 | Dnmt1 | 0.0009739 |
| chr3:79769701-79772600 | NM_001000294 | Olr461 | 0.0009739 |
| chr8:76648601-76652000 | NM_053345 | Gtf2a2 | 0.0009739 |
| chr3:84072901-84075600 | NM_001000347 | Olr664 | 0.0009739 |
| chr14:5092201-5095600 | NM_001008338 | Lrrc8d | 0.0009739 |
| chr10:90897601-90900500 | NM_148891 | Nmt1 | 0.0009739 |
